# The Secular Press on the Treatment of Yellow Fever

**Published:** 1878-10

**Authors:** 


### THE SECULAR PRESS ON THE TREATMENT OF YELLOW FEVER.

We have not been surprised to notice in the columns of the daily press, criticisms upon the method and apparent want of success of the treatment pursued by our medical brethren, who are heroically contending against the epidemic of yellow fever in several of the Southern States. These criticisms have elicited, in one instance at least, a protest on the part of a well-known physician of this city, which has been accorded that degree of reserved acceptance usual in such cases. Daily journals are not given to unreserved acknowledgment of error. Their presses, like the king, "can do no wrong."

We say that we have not been surprised, simply because every intelligent man has learned the exact value of newspaper editorial opinions in this country. Upon any subject whose essential facts are recorded in text-books, census and government reports, biographies, and encyclopœdias—the average library of the "sanctum"—these writers speak by the card. In the small hours of the morning, when presses are already in motion, boys calling for "copy," and a thousand readers are waiting for an edition, the results of a superficial reading upon any given subject, dressed in the English of the average penny-a-liner, are often really surprisingly clever. He, however, who has completely mastered any subject thus discussed, smiles at the inaccuracy of the premises and the feeble shallowness of the conclusions reached.

They do these things differently in England. There, editorial articles (generally called "leaders") are carefully written, often weeks in advance, by men who have been specially prepared by years of study in the department alloted to their charge—men who often live at a distance from the metropolitan centers.

What we have to say upon the subject of yellow fever is already known to the world of science; it is well, however, to occasionally recite old truths.

Physicians are called to encounter three classes of disease; first, those which terminate favorably without human intervention; second, those which terminate fatally if neglected, and favorably if judiciously treated; third, those which terminate fatally in any event. Some diseases exhibit varieties in each class. Such a disease is yellow fever.

Mild cases of yellow fever, terminating favorably without medical treatment, occur in this as in all other epidemics. The fact is often best illustrated in the phenomena of the malady displayed in the negro race, though by no means confined to this class. Naturally these cases attract but little attention in the public press.

Other cases there are which, under almost any wise and judicious treatment, go on to recovery. Strange as it may sound to those unfamiliar with the facts, these cases outnumber all others, except in the very foci of contagion, and in the most malignant of epidemics. This year they can be numbered by the thousand, but patients in complete convalescence possess no interest in the eyes of the average newspaper writer and newspaper reader. It is the house which is in process of demolition that attracts the crowd, not the structure that is in building.

Nor is the treatment of these cases essentially peculiar. The principles of treatment which we recognize as based upon a sound pathology and large experience, will guide almost any judicious practitioner in the right course. There is no cast-iron rule for such cases. With a mortality of ten or fifteen per cent. of all patients seized with the malady, it is evident that some latitude can be allowed to those who are fitted by skill or experience to deal with the plague.

Lastly, there are the cases in which treatment is of no avail, and under any aspect, the issue of these is terrible. Patients of this sort are hopelessly doomed from the instant of attack, and the attack is remorselessly swift and direct. Any treatment worth the name, should have equal swiftness and intensity, but while the world lasts, none such will be found. For patients of this class are either, at the outset, stricken down with the virulence of the malady or, having been gradually and insidiously poisoned till the point of saturation is reached, they suddenly succumb.

The so-called "walking cases" are to be explained in this way, those in which a man who is engaged in the street, or in the counting-room, unexpectedly ejects black vomit and dies. Even in such persons, however, a skilled eye can early recognize symptoms of a formidable and even of a fatal character, which elude the observation of the less vigilant. Their hour of actual dissolution may be deferred for a brief space of time, but they are nevertheless dying at the earliest onset of the malady—death is occurring at the kidneys, whose function is suspended so suddenly that no opportunity is given for the usual protest of nature; it is occurring in the saffron-tinted skin, which often assumes the peculiar hue suggestive of incipient moist gangrene—it is occurring at the stomach whose black ejecta (even including the so-called "flies' wings" appearance), can be seen occasionally in other diseases of rapidly fatal prostration. In short, what typhoid and typho-malarial fevers can accomplish in weeks, yellow fever can accomplish in as many minutes.

When the heart of a man is perforated by a bullet, no one expects the surgeon to "cure." When a despairing wretch has swallowed a dose of strychnia, and the deleterious drug or its newly-formed compounds, have been absorbed from the stomach, society exonorates the physician from a share in the fatal result. If the bullet spare a vital organ, if the poison can be reached by pump or antidote, a responsibility is incurred by the practitioner.

But even surer than the bullet or the chemical salt is the intensest poison of yellow fever when it has once gained access to the human economy. Its full possession of the system is equivalent to doom. In such a person medical skill is utterly helpless.

We who write these lines know whereof we are writing. We have been in the midst of an epidemic, watched its career, despaired over its victims, saved those that were savable, received its foul ejecta in the face, and made post mortem examinations of its dead. We are not, therefore writing in the phrases of the press-room, but in the language of experience.

What medical skill can do in other morbid states of the human economy, that it can, and actually does accomplish in the treatment of yellow fever. What it can not do, when a ball has pierced the brain, a virus permeated the blood-mass, that it cannot do in yellow fever. Yet in the one case the newspapers complain and in the other they do not.

The search, nevertheless, for the philosopher's stone, the elixir of life and the drug that can work miracles, will be continued. The greatest sinner in his moment of penitence can be shrived by the word of a priest. Why then cannot a man put a pill into his stomach that will absolve him from the consequences of the poison he has ingested, absorbed or inhaled to his mortal hurt? This is the argument of the world.

We know it cannot be done. We of the medical profession, who have studied, observed, reasoned and refused to believe in modern miracles, know that no plant that grows, no mineral in the earth, nothing that burrows, flies or swims, will ever give absolution in these cases. A larger knowledge may enable us to push the advanced line of savable cases further into the domain of the unsavable; it may give us a better prophylaxis. But while a man will burn his hand who holds it in the fire, so long will human life shrivel like a dried leaf in the full breath of this pestilence.

Our medical brothers in the South have built for themselves a monument more enduring than brass. Of the lives saved by their self-sacrificing devotion, there is no human record. Many of them have set on high before us an example of sublime heroism which can never be lost.

---

The Roll of Honor.—The following is an incomplete list of the noble dead who have fallen in the battle against yellow fever in the South: Drs. Chas. Gallagher, Herndon, B. W. Avant, Woolfolk, Chas. Bonner, Catlett, T. L. Bond, Menees, J. R. Renner, Jno. B. Hicks, Lehman, Jno. Erskine, Cage, McGregor, Blanton, Milton, Hawkins, J. S. Bankson, W. W. Hall, Hughes, Gillespie, E. J. Hughes, May, F. Sarner, J. C. Rogers, Hopson, K. T. Watson, W. R. Hodges, T. M. Dickinson, R. B. Williams, Mead, Booth, P. F. Whitehead, J. G. Byrne, Nathan McGee, J. E. Penn, M. J. McKee.

